# Protocol: A mixed methods evaluation of an IPS program to increase employment and well-being for people with long-term experience of complex barriers in Vancouver’s downtown and DTES

**DOI:** 10.1371/journal.pone.0261415

**Published:** 2021-12-16

**Authors:** Amanda Kwan, Jonny Morris, Skye P. Barbic

**Affiliations:** 1 Department of Occupational Science and Occupational Therapy, Faculty of Medicine, University of British Columbia, Vancouver, BC, Canada; 2 Canadian Mental Health Association BC Division, Vancouver, BC, Canada; 3 Providence Health Care Research Institute, Vancouver, BC, Canada; Xiamen University - Malaysia Campus: Xiamen University - Malaysia, MALAYSIA

## Abstract

**Background:**

Employment improves mental health and well-being by providing financial security, daily structure, a sense of identity and purpose, and social engagement. However, securing and sustaining employment is exceptionally challenging for vulnerable populations who experience persistent and multiple barriers, such as mental illness, homelessness, food and housing insecurity, and marginalization. Evidence-based supported employment programs, most notably individual placement and support (IPS) are becoming a more common approach for addressing the needs of these high-risk individuals. The aim of this paper is to outline the protocol for evaluating an IPS program in Vancouver’s downtown and Downtown Eastside (DTES).

**Methods and design:**

This prospective quasi-experimental study of persons with persistent and multiple barriers to employment will use a mixed-methods approach for evaluating a novel IPS program. The evaluation will consist of survey packages and interviews that will capture outcomes related to employment and well-being, as well as the experiential process of receiving individualized and integrated supports through the IPS program. A mixed-methods approach is appropriate for this study as quantitative data will provide an objective assessment of program impacts on employment and well-being outcomes over time, while qualitative data will provide an in-depth understanding of continued barriers and experiences.

**Discussion:**

The results from this evaluation will contribute evidence within a local British Columbian (BC) context that may increase access to meaningful employment for those with long-term experience of complex barriers to employment. Further, the findings will support continued improvements, and guide decision-making around practices and policy for future implementation of IPS and employment supports across BC.

## Introduction

Unemployment rates in British Columbia (BC), Canada have risen from 4.7% in 2019 to 7.5% at the start of 2021 [1, 2]. Although unemployment rates have slowly started to decrease towards the end of the year (5.9%), there still remains over 160,000 people in the province struggling to secure employment [3]. Sustained unemployment is associated with a number of negative health and social outcomes, including financial hardship, labour market detachment, social deprivation, psychological stress, mental health concerns, and mental illness [1, 4, 5]. Conversely, sustained employment can improve quality of life and satisfaction with life by providing individuals with structure, security, a sense of purpose, and opportunity for increased social support and inclusion, which collectively contribute to improved mental health and stability [4–6]. Moreover, studies consistently show that despite experiencing numerous challenges and barriers to employment, the majority of people with mental illness want to be working [7–10]. In response, supported employment programs have continued to expand throughout North America and worldwide [11].

Supported employment refers to the assortment of programs and services available to help people with disability secure competitive work [7]. Individual placement and support (IPS) is an evidence-based supported employment model targeted specifically to help people with severe mental illness or disability obtain and sustain employment [12–14]. To date, IPS programs are seeing greater employment outcomes compared to traditional vocational rehabilitation across populations with varying mental illnesses, such as those experiencing depression, PTSD, psychosis, and/or schizophrenia [6, 7, 15–18]. IPS programs are also seeing greater employment outcomes across different subpopulations, such as with veterans, youth, and individuals experiencing or at-risk of homelessness [15, 19, 20]. In addition to improved employment outcomes for this population, there is evidence to suggest that IPS programs also benefit a variety of secondary outcomes related to quality and duration of employment [17]. However, there is recognition that a fuller range of outcomes needs to be investigated given that those who experience mental illness are also likely to experience a range of other physical, social and socioeconomic barriers that impede the likelihood of sustainable employment.

One of the core principles of the IPS model is the intentional integration of employment services with broader mental health treatment [12]. In Canada, the first point of contact for someone struggling with mental health problems is commonly their primary care provider [21]. At this time, it is not typical that employment status is a priority in the primary care setting, despite the positive health and social outcomes associated with sustained employment [22]. By integrating employment services directly within primary care, patients may receive convenient access to a variety of healthcare and social service professionals, such as family physicians, nurse practitioners, psychologists and mental health specialists, occupational therapists, and employment specialists, who can work collaboratively to support their overall well-being. Although this integrated approach in primary care is promising for supporting populations with mental illness, we are unaware of any research in this area, notably within the Canadian context [22]. Opportunity exists to understand the effectiveness of IPS programs embedded within primary care to address the health, social, and employment goals of this population, including those who may experience other complex barriers such as food and housing insecurity, poverty, trauma, and low education and literacy.

In British Columbia, individuals who face a disproportionate number of barriers to employment may receive a designation by the Government as “persons with persistent and multiple barriers (PPMB).” Individuals with PPMB status have a persistent health condition, and may experience any combination of other health and social challenges including but not limited to homelessness or risk to homelessness, food insecurity, unsafe housing, and marginalisation, as well as need basic skills or language training for employment readiness [23]. A health condition may be physical or mental, which includes any and/or a combination of depression, anxiety, PTSD, schizophrenia, bipolar disorder, and neurodivergence. Although the current body of evidence supporting IPS has led to an increased call in the province to provide IPS programs to help support our most vulnerable populations [24], how best to integrate and evaluate this service within existing health services is unknown.

### Study objectives

Our overarching objective is to contribute new knowledge of IPS embedded within primary care to inform decision making, and guide practice and policy related to employment supports for vulnerable populations (e.g., individuals with PPMB status) across British Columbia. Specifically, our research aims are to:

1. Assess the effectiveness of the IPS program for securing employment for individuals with PPMB status;
2. Assess the impact of individualized and integrated primary care supports provided through the IPS program on health and social outcomes for individuals with PPMB status; and
3. Gain an understanding of the experiential process of the IPS program from individuals accessing the program, as well as from healthcare professionals and staff involved in its implementation.

## Materials and methods

This is an ongoing prospective quasi-experimental study that will run for 18 months from February 2021 to September 2022. The evaluation is designed as a mixed-methods process that will assess the impact on employment, health, and social, outcomes of the *Links to Employment* program provided through the Canadian Mental Health Association (CMHA) BC Division. In addition, this evaluation will investigate the implementation process of the IPS program, in order to improve program features and practices for future implementation, including anticipated program expansion. All components of this study have been approved by the University of British Columbia Behavioural Research Ethics Board (BREB) on March 17, 2021 (UBC BREB# H20-02198).

### Intervention

*Links to Employment* is a novel supported employment program targeted at promoting the well-being of vulnerable individuals within Metro Vancouver, British Columbia, a diverse city of 2.6 million people located on Canada’s west coast. Within Vancouver’s downtown core exists a neighborhood called the Downtown Eastside (DTES), often described as a community with a complex set of social issues including high levels of substance use, homelessness, poverty, and crime [25]. Our intervention includes those living in the DTES. Key features of the *Links to Employment* program include time unlimited services, access to IPS trained and trauma informed program staff embedded within a primary care team, involvement of an integrated care team that includes clinicians located within the same primary care setting, and ongoing focus on health and well-being simultaneous to vocational support. Continued consideration of environmental needs (e.g., safe housing), and mental and emotional well-being is also a critical component, given the vulnerability of the target population. By engaging a variety of professionals in a coordinated way, including a vocational counsellor, occupational therapist, job developer, case manager, and primary care providers, the program is able to provide greater integrated health services and supports to support individualized employment and health goals.

**Fig 1:**
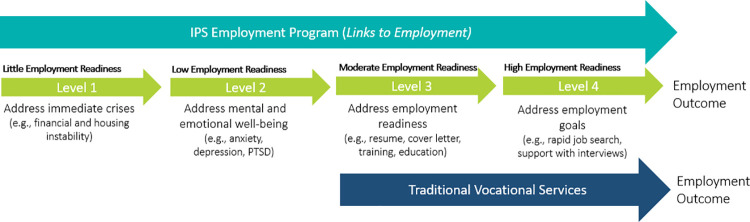
Intervention flowchart.

The *Links to Employment* program is designed to support complex populations, primarily those who currently have little to low employment readiness, throughout their entire employment journey. This remains an unmet need for a large portion of those living or accessing services in the DTES. For this population, existing vocational services (traditional) targeted to those with moderate to high employment readiness are not sufficient or effective. Fig 1 below highlights the flow of services/supports within the IPS program compared with traditional vocational services available in British Columbia, and illustrates the gap in services/supports *Links to Employment* is currently filling.

### Sites

The *Links to Employment* IPS program is operating out of two central sites located within primary care settings: The Three Bridges Community Health Centre located in Vancouver’s central downtown area and the REACH Community Health Centre located adjacent to Vancouver’s DTES.

### Participants

Participants are eligible for this study if they are receiving or will be receiving employment supports through the IPS program. This includes individuals currently enrolled in the program, as well as those waitlisted. As such, eligibility is primarily based on eligibility criteria being met for enrollment in the IPS program; there is no other exclusion criteria for this study. To receive a referral to the program, all individuals must be considered eligible for PPMB status by a primary care physician. That is, individuals need to currently be experiencing a number of barriers to employment that are not expected to improve in the short-term. Qualifying barriers include, but are not limited to, a persistent health condition, homelessness or risk to homelessness, the need for basic skills or language training, a criminal record, food insecurity, unsafe housing, discrimination, and marginalisation [23]. In addition to meeting the above criteria, study participants must also be English speaking adults between the ages of 19–64 years old. Study components are only available in English.

In addition to those enrolled in the IPS program, all healthcare providers and staff involved in the implementation of the program will be invited to participate in the research by providing their feedback and experience around the implementation process.

### Recruitment

To ensure consistency in recruitment, we have developed a recruitment protocol to engage clients in the IPS program for the research study. The initial introduction to the study is made through IPS program staff (vocational counsellor, occupational therapist or program manager), who provide a brief summary of the study to their clients and seek permission for them to be contacted by research staff. If clients are interested, program staff also provide a copy of the detailed study information and blank consent form. Program staff them contact the research coordinator, who will contact clients via their preferred method of communication (e.g., email, text, phone) within 1 week. All potential participants have multiple opportunities to engage with the research coordinator prior to providing their signed consent. A traditional (print) or digital signature is accepted.

### Sample size

Sample size of participants will depend on the capacity of the IPS program, and client turnover within the program. Based on IPS fidelity standards [26], we anticipate a 30-client capacity at any given time, as well as a 30-client waitlist. We expect to engage with a subset of these groups, enrolling 10–20 participants from the program and a comparable number of those waitlisted.

### Safety

Given the observational nature of this study, there are no direct risks associated with participation, as individuals will continue along their natural employment service trajectories. While some of the questions included in the assessments may trigger negative feelings or discomfort for participants, we attempt to mitigate this by providing a list of local mental health resources and contacts alongside each assessment, as well as collaborating with their broader care team, as necessary.

### Data collection

Given the complexity of the study population, current COVID-19 pandemic public health restrictions, and diverse nature of the research questions, a mixed-methods approach will be used for data collection. The quantitative assessments will be used to capture demographic characteristics about the population enrolled and waitlisted for the IPS program, as well as objectively measure their employment, health, and social outcomes over time. The qualitative assessments will be used to capture a more in-depth and detailed understanding of the experiential process, including program feedback, from those who received services/supports through the IPS program, as well as those involved with implementation. Drawing from multiple sources for data is a form of methodological triangulation that allows us to investigate different but complementary aspects of a phenomenon to gain a more exhausted understanding of it [27, 28]. For example, through quantitative data we may learn that a participant’s levels of anxiety has steadily decreased over time. Through the complementary qualitative data, we may learn what factors (e.g., access to mental health services, increased social network of supports, steady employment, and/or housing stability) have contributed the most to the decrease in anxiety seen.

**Fig 2:**
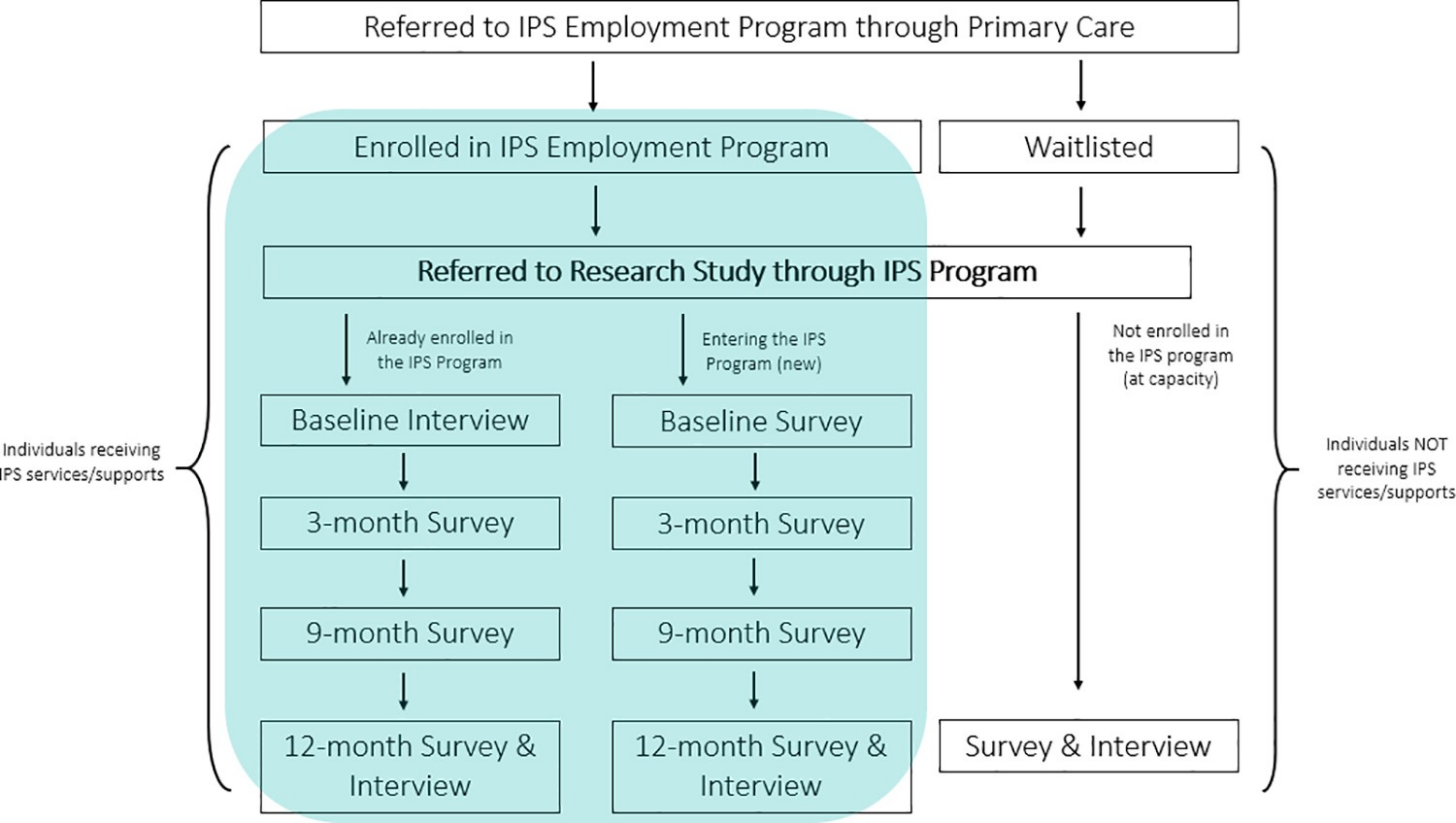
Study flowchart (individuals referred to the IPS employment program).

Fig 2 below illustrates the study flowchart. Participants will be assessed at 4 points in time over the course of one year (baseline, 3-months, 9-months, & 12 months). These assessments include a combination of surveys and interviews, dependent on whether a participant is already enrolled in the IPS program and has been receiving services/supports at the start of data collection or whether a participant is just entering the IPS program. For those already enrolled in the program, a baseline interview will be conducted by the research coordinator to allow participants to reflect on their experiences to date, as well as provide estimated measures of their baseline well-being (i.e., when they first entered the program). Individuals new to the program will complete a baseline survey package consisting of 6 questionnaires that assess their general demographics, employment barriers, and health and social well-being. After baseline assessments, all participants enrolled in the study will complete the same research activities at their 3-month, 9-month, and 12-month assessments. Should a participant elect to exit the IPS program after securing employment or for any other reason, they will continue to be assessed as part of the research study, following the same trajectory of activities. Participants who elect to leave the IPS program and the research before the completion of one year will be offered the end-of-study interview to ensure capture of their experiences to date. Should a participant be loss to follow-up, we will be transparent in our data reporting and address the challenges and limitations when working with vulnerable and hard-to-reach populations in all publications, reports, and knowledge translation materials.

In addition to data collection from those who participated in the IPS program, a subset of individuals who remain on the waitlist at 12 -months will complete an assessment of their current employment and well-being. All program participants will be compensated for their time, receiving an honorarium for each research activity completed up to a total of $200.00 Canadian.

Service providers involved in the implementation of the IPS program will complete an open-ended survey. There will be no compensation for this activity.

#### Demographic data

Data on socio-economic characteristics will be collected as part of the baseline assessment. This information includes but is not limited to age, sex at birth, gender, level of education, language(s) spoken, ethnicity, self-reported health condition, and current living situation.

#### Employment outcome

The primary outcome of interest is the proportion of study participants who secure employment over the course of one year. In addition to this, employment quality and sustainability will be assessed through both quantitative survey questions and qualitative interview questions. Participants will be asked to provide detailed information about their employment/training (e.g., hours of work, nature of work, wage) and self-rate their employment satisfaction, as well as have the opportunity to provide their in-depth experiences and reflections around their current or past employment.

#### Health and social outcomes

Secondary outcomes of interest include measures of both health and social well-being over time. The GAD-7 [29], PHQ-9 [30], C-PROM [31], ReQoL-10 [32], and SWL-5 [33] are all established questionnaires, with established psychometrics within this population, that will be included as part of each survey package. Each questionnaire provides unique insight into the health and well-being of an individual: the GAD-7 and PHQ-9 are screeners for anxiety and depression respectively; C-PROM (30 questions) is a measure of client recovery; ReQoL-10 is a measure of quality of life specifically for people with mental health challenges; and SWL-5 is a measure of overall life satisfaction.

#### Qualitative data

To complement the employment, health, and social outcome data described above, qualitative data will be collected from both 1:1 semi-structured interviews (program participants) and open-ended survey questions, including questions about how PPMB status was determined (service providers). Program participants will have the option to complete their interview in-person, over the phone, or through the video conferencing system Zoom. Service providers will complete their survey electronically. The decision to use an open-ended survey rather than conduct interviews with service providers was based on the anticipated time commitment. The selected approach allows for capture of in-depth qualitative feedback with minimum disruption to workflow. Both instruments were developed based on existing evaluations for IPS and the IPS model, and discussed across a multi-disciplinary team to ensure contextual appropriateness.

The qualitative data collected will provide a better understanding of how the IPS program was experienced and key strengths/weaknesses to its implementation, as well as continued barriers/gaps in supports. Sample questions/prompts from the interview guide for program participants include: Describe your experience with the *Links* employment program; what key factors or supports or needed to help you achieve your short/long-term employment goals?; what do you feel are necessary supports for maintaining employment?. Sample questions/prompts from the open-ended survey for implementation staff include: In your opinion, what key factors are needed to maximize the employment/education potential of individuals with PPMB status over the long term?; how would you describe the implementation of this IPS program?.

### Data analysis

Quantitative data will be analyzed using version 4.0.2 of R software: A language and environment for statistical computing [34]. Descriptive statistics will look at the mean (SD) or median (IQR), and frequency of demographic characteristics, and employment, health, and social outcomes. In addition, Chi-squared tests and independent-sample t-tests will be performed (sample size permitting) to compare demographic variables such as gender and employment status for those who received IPS program services/supports compared to those who did not after one year (waitlisted), and paired t-tests or Wilcoxon signed rank test to compare health and social outcome measures for program participants. Outcome measures at each of the 3 points in time following baseline (3-, 9-, 12-month) will be compared against baseline and the previous timepoint to determine if there is a significant difference.

Qualitative data will be analyzed using version 12 of NVivo Pro [35]. Interviews will be audio recorded and transcribed with participant consent, and thematically analyzed using an inductive approach [36]. This will allow themes and significant ideas/concepts around barriers/facilitators and experiences to emerge from the raw data. All analysis will be performed by at least two researchers (AK, SB) to ensure consistency and minimize individual bias. The emergent codebook (node structure) will support a more deductive approach in future assessments of this program.

## Discussion

There is widespread recognition and a strong body of evidence that IPS is an effective approach for improving employment outcomes for those with severe mental illness [6, 7, 15–18]. However, there is limited literature on IPS within the BC or Canadian context, and integrating this as a core service within health to support complex vulnerable populations with both their health and employment goals. The majority of supported employment studies are based in the US and Europe, where different systems and infrastructures for care exist [11, 17]. Additionally, the majority of studies have focused exclusively on those afflicted by a specific mental illness (e.g., psychosis, schizophrenia, PTSD, etc.), rather than considering the broad spectrum of mental illnesses that may be present in a high-risk and vulnerable subpopulation, the context in which they receive and access services, and their personal goals and needs. This study is inclusive of any mental illness or combination of mental illnesses and considers changes in health and well-being an important indicator of employment readiness and future employment success. As such, this study includes a variety of interim outcomes not related to employment but rather employment readiness or general well-being (e.g., anxiety and depression, quality of life, satisfaction with life, community engagement and social networks, continued employment barriers). Lastly, while supported employment programs are often integrated into a variety of community settings (e.g., mental health centres, rehabilitation programs, clubhouses) [7], this study will evaluate an IPS program embedded within primary care settings, which we hypothesize will allow for more intentional integration of healthcare and employment teams, to work together in a systematic, person-centred, and strength-focused perspective.

There are potential limitations to this study protocol. First, the study is quasi-experimental rather than randomly controlled. Given the known needs of the population in Vancouver’s downtown and DTES, and pilot RCT work led by our team, there were ethical considerations around assigning high-risk individuals to traditional vocational services (e.g., WorkBC) that are not designed for people with severe mental illness [37], and proved harmful to mental well-being during an initial pilot. Second, measures of fidelity are not included as part of this evaluation as the program is newly developed and there continue to be revisions to its structure, staffing, and implementation. Given the correlation between high fidelity and employment outcomes [11, 26], fidelity reviews will be built into the evaluation process for future assessments. For the time being, our focus is on understanding the implementation process and organizational necessities for successful adoption of IPS. A number of implementation barriers that cut across the levels of government (e.g., sector fragmentation, funding), organization (e.g., lack of education about the model and leadership buy-in), and program administration (e.g., resistant to change, undertrained) have already been recognized [7, 38]. Through this study, we hope to better understand the experiences of those directly involved with the program and its delivery, to identify and address any implementation barriers that may be applicable within our local BC context.

The results of this study will be disseminated through a variety of different mediums targeted to our range of stakeholders (e.g., CMHA Research and Clinical Steering Committees, Vancouver Coastal Health, relevant Province of BC Ministries) and audiences (e.g., community/general population). Knowledge translation activities include but are not limited to academic publications and conference presentations, community and government reports, community engagement activities including World Cafés, and traditional medial/social media activities.

In conclusion, the novel findings from this research will provide insights into the impact and effectiveness of IPS embedded within primary care. This will contribute to ongoing strategies and practices for addressing the employment and well-being needs of British Columbia’s most complex and vulnerable population. Additionally, findings will be used to inform decision making and guide policy around employment supports at local, provincial, and federal levels.
